# Context‐dependent effects of whole‐body vibration on reproductive function and penile tissue integrity in sedentary male rats

**DOI:** 10.1113/EP094086

**Published:** 2026-10-02

**Authors:** Mujdat Ayva, Senay Akin, Irem Gungor Orhan, Neslihan Coskun Akcay, Berkay Ozerklig, Kemal Kosemehmetoglu, Mesut Altan, Haydar A. Demirel, Fazil Tuncay Aki

**Affiliations:** ^1^ Department of Urology Hacettepe University Faculty of Medicine Ankara Turkey; ^2^ Division of Exercise and Sport Physiology, Faculty of Sport Sciences Hacettepe University Ankara Turkey; ^3^ Department of Obstetrics and Gynecology Hacettepe University Faculty of Medicine, In‐Vitro Fertilization Unit Ankara Turkey; ^4^ Department of Pathology Hacettepe University Faculty of Medicine Ankara Turkey; ^5^ Center for Sport Sciences Near East University Nicosia Cyprus

**Keywords:** sedentary behaviour, spermatogenesis, whole body vibration

## Abstract

Sedentary behaviour is increasingly recognized as a risk factor for systemic disorders; however, its effects on male reproductive health and penile tissue integrity remain insufficiently understood. Furthermore, the therapeutic role of whole‐body vibration (WBV) has not been investigated. This study evaluated the effects of sedentary behaviour on testicular and penile tissues and examined whether WBV could attenuate these alterations. We hypothesized that sedentary behaviour would impair spermatogenesis and penile tissue integrity, whereas WBV would attenuate these effects. Thirty‐two male Wistar rats were randomized into four groups (*n* = 8/group): Control, Sedentary, Sedentary+WBV and WBV. Sedentary conditions were induced for 28 days via a reduced‐cage model, with daily WBV application (45 Hz, 3 mm). Serum testosterone, sperm parameters, testicular histology and penile smooth muscle‐to‐collagen ratio were assessed. Sedentary behaviour impaired spermatogenesis, with lower Johnsen scores (*P* = 0.008), reduced sperm concentration (*P* = 0.006) and motility (*P *< 0.001), and increased morphological abnormalities (*P *< 0.001). It also reduced the penile smooth muscle‐to‐collagen ratio (*P *< 0.001), indicating adverse structural remodelling in erectile tissue. In sedentary animals, WBV attenuated several alterations, preserving sperm quality and cavernosal smooth muscle content. However, WBV alone impaired spermatogenesis and increased sperm morphological abnormalities in non‐sedentary animals. Serum testosterone levels did not differ significantly. In conclusion, prolonged sedentary behaviour impaired male reproductive indices and induced adverse structural remodelling of penile tissue in male rats. WBV showed context‐dependent effects, attenuating alterations under sedentary conditions while showing adverse associations in non‐sedentary animals. These findings warrant further translational investigation into the context‐dependent effects of WBV on male reproductive and penile tissues.

## Abstract (highlights)

- **What is the central question of this study?**
  Does sedentary behaviour impair testicular histology, sperm parameters and penile tissue integrity? Can whole‐body vibration (WBV) serve as a therapeutic intervention to mitigate these adverse alterations?
- **What is the main finding and its importance?**
  Sedentary behaviour significantly impairs spermatogenesis and induces adverse structural remodelling in penile tissue. This study provides the first evidence of the ameliorative effects of WBV on spermatogenesis and penile structure under sedentary conditions. Conversely, WBV applied under normal, non‐sedentary conditions exerts adverse effects. This highlights the highly context‐dependent role of WBV.

## 1 INTRODUCTION

Sedentary behaviour is characterized by spending a substantial proportion of waking time in low‐energy activities, such as sitting, reclining or lying down, with an energy expenditure of ≤1.5 metabolic equivalents (METs) (Bull et al., 2020; Tremblay et al., 2017). Owing to technological advances and the demands of contemporary living, sedentary behaviour has become increasingly prevalent worldwide. Prolonged sedentary behaviour has been associated with several adverse health outcomes, including cardiovascular disease, obesity, type 2 diabetes and metabolic syndrome (Owen et al., 2010; Tremblay et al., 2017).

Given these multisystem effects, increasing attention has been directed toward the possible impact of sedentary behaviour on male reproductive health and penile tissue integrity. However, much of the current literature has addressed this issue primarily through the framework of physical inactivity, despite evidence that sedentary behaviour represents a distinct behavioural construct. Unlike physical inactivity, which refers to insufficient participation in moderate‐to‐vigorous physical activity, sedentary behaviour is characterized by prolonged periods of sitting or reclining and may exert adverse biological effects even among individuals who meet current physical activity recommendations (Biswas et al., 2015; Patterson et al., 2018).

Although regular exercise is a well‐established strategy for improving health, it may not always be practical for desk‐based workers, older adults or individuals with limited mobility. Therefore, alternative low‐effort interventions that may counteract the biological consequences of prolonged sedentary behaviour merit investigation. Whole‐body vibration (WBV) has emerged as one such approach. WBV induces low‐level muscle activation through mechanical oscillations delivered via specially designed platforms and has been reported to influence cardiovascular, musculoskeletal, endocrine and neurological systems (Bonanni et al., 2022; Cardinale & Bosco, 2003; Chanou et al., 2012). Despite these observations, experimental evidence regarding the effects of WBV on male reproductive and penile health remains limited, particularly in the context of sedentary behaviour.

Previous experimental studies using restraint‐based models have reported impaired spermatogenesis and sperm quality, although these models primarily induce physiological stress rather than sedentary behaviour (Arun et al., 2016; Mehfooz et al., 2018). Likewise, occupational and experimental studies have suggested that WBV may influence male reproductive function, with prolonged or occupational exposure associated with impaired spermatogenesis, reduced sperm quality, and molecular changes related to oxidative stress and inflammation (Krajnak et al., 2022; Zarei et al., 2022). However, these studies did not examine WBV as a potential intervention under sedentary conditions. Therefore, whether WBV attenuates or exacerbates sedentary behaviour‐induced alterations in male reproductive and penile tissues remains unknown. Accordingly, the primary aim of the present study was to investigate the impact of prolonged sedentary behaviour on male reproductive function and penile tissue integrity and to evaluate the potential role of WBV in attenuating these alterations. We hypothesized that sedentary behaviour would impair spermatogenesis and sperm quality and induce structural deterioration of penile integrity, whereas WBV would attenuate these adverse alterations under sedentary conditions.

## 2 METHODS

### 2.1 Ethical approval

All procedures were approved by the Hacettepe University Local Ethics Committee for Animal Experiments (Approval No. 2022/53) and were conducted in accordance with the national regulations governing the welfare and protection of animals used for experimental and other scientific purposes, as well as the *Guide for the Care and Use of Laboratory Animals* (8th edition, 2011).

### 2.2 Animals

Thirty‐two adult male Wistar albino rats aged 3–4 months and weighing 300–320 g were acclimatized for 1 week under specific pathogen‐free conditions (21 ± 2°C, 50%–60% humidity, reversed 12‐h light/dark cycle) with ad libitum access to standard chow and water.

Following a 1‐week acclimatization period, animals were randomly assigned to four groups (*n* = 8 per group), and the 28‐day sedentary intervention and whole‐body vibration protocols were initiated immediately thereafter. The control group (C) remained in standard cages without intervention. The sedentary group (S) was housed in custom cages designed to restrict movement and induce sedentary behaviour, as previously described (Gungor‐Orhan et al., 2025; Marmonti et al., 2017). The sedentary + WBV group (SV) was exposed to the same conditions and received daily WBV. The WBV group (V) was housed in standard cages and received daily WBV, allowing evaluation of WBV in the absence of sedentary conditions (Figure 1).

**FIGURE 1:**
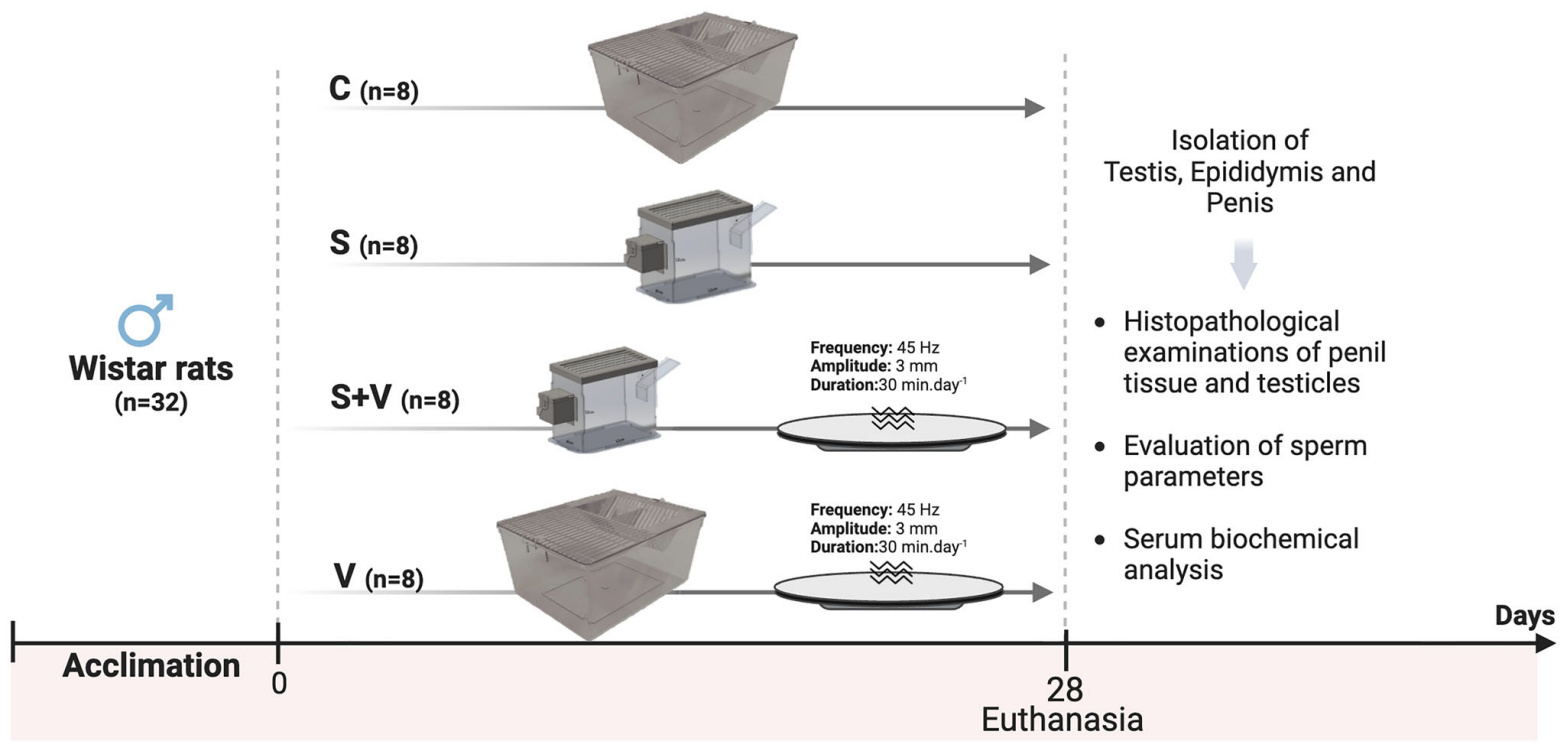
Schematic overview of the experimental design. C, control; S, sedentary; V, whole‐body vibration; SV, sedentary + whole‐body vibration.

### 2.3 Experimental sedentary behaviour model

Sedentary behaviour was modelled using reduced cage space, which has previously been reported to limit living area without causing physiological stress (Gungor‐Orhan et al., 2025; Marmonti et al., 2017). Animals in the sedentary groups were housed in custom‐made Plexiglas cages (12 × 12 × 8 cm), corresponding to approximately 20% of the volume of a standard cage. These cages restricted locomotor activity while allowing free access to food and water. Ventilation was provided through a grille on the cage roof. Bedding was replaced twice daily, and animals were briefly transferred to identical pre‐prepared cages during cleaning in order to maintain consistent movement restriction (Figure 2).

**FIGURE 2:**
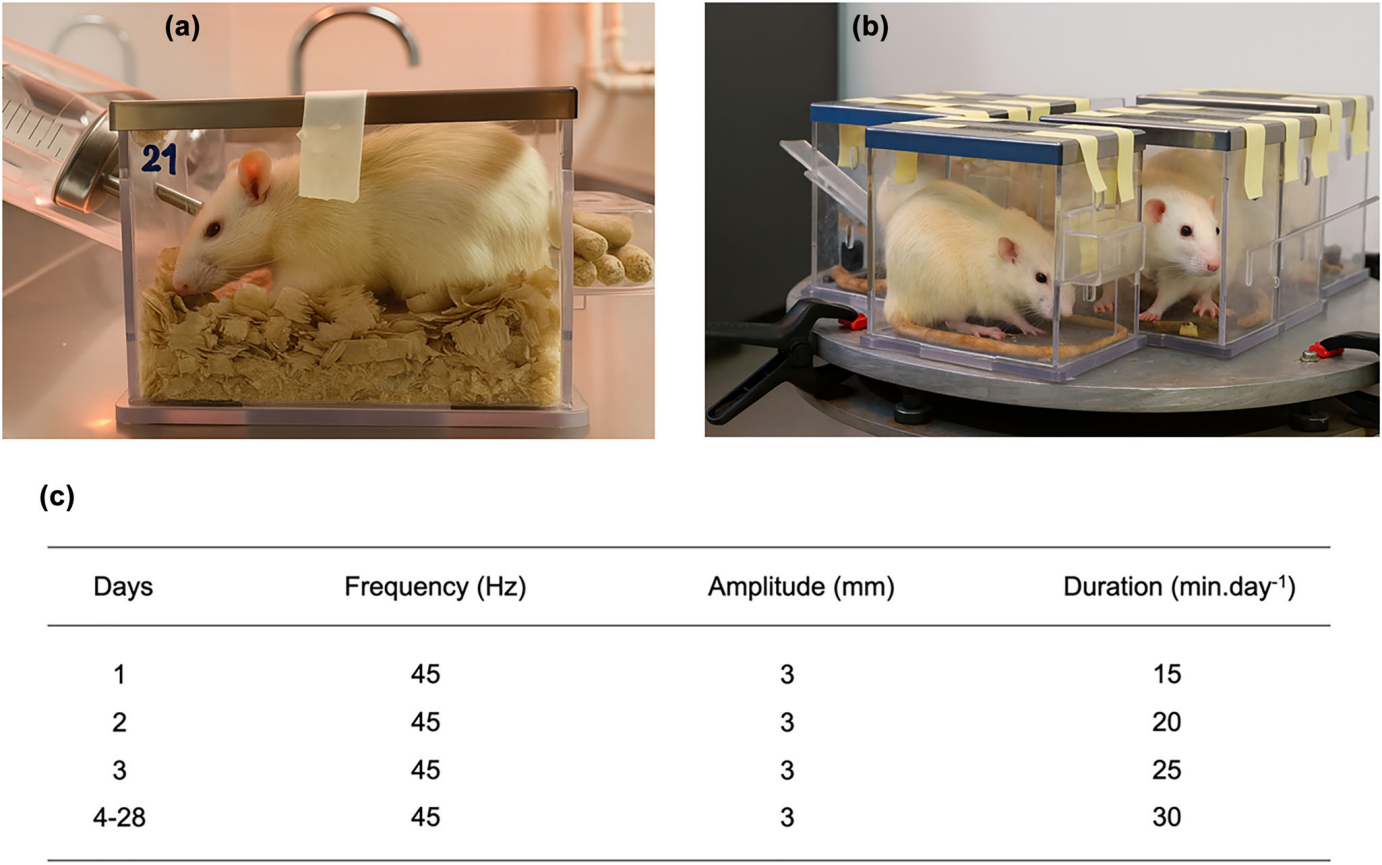
Experimental setup and whole‐body vibration (WBV) protocol used in the study. (a) Reduced‐cage volume model. Custom‐designed cages (12 × 12 × 8 cm) were used to restrict voluntary movement and induce sedentary behaviour. Rats in the S and SV groups were housed in these cages for the duration of the experimental period. (b) Whole‐body vibration (WBV) application. The vibration platform operated at a frequency of 45 Hz and an amplitude of 3 mm, allowing rat cages to be placed securely and stably. Rats in the SV group were placed on the platform while remaining inside their restricted cages, whereas rats in the V group were positioned directly on the platform without movement restriction. (c) WBV protocol. WBV was administered once daily, with session duration progressively increased during the initial days (15–30 min/day) and maintained at 30 min/day from day 4 to day 28.

### 2.4 Whole‐body vibration protocol

WBV was delivered using a custom‐designed mechanical platform set to a target frequency of 45 Hz and an amplitude of 3 mm. Each WBV session lasted 30 min/day. Animals in the SV group were placed on the platform while remaining in their restricted cages, whereas animals in the V group were placed directly on the platform under standard housing conditions. Sessions were conducted daily, 4–5 h after lights‐off, during the animals’ active phase. To facilitate adaptation to the vibration stimulus, session duration was progressively increased during the first three days (15–30 min/day) before reaching the target duration of 30 min/day for the remainder of the intervention (Figure 2).

### 2.5 Euthanasia and tissue collection

At the end of the study, following the final intervention, rats were deeply anaesthetized with ketamine (90 mg/kg) and xylazine (10 mg/kg) during the dark phase (09.00–12.00 h). Blood samples were collected from the descending aorta, after which the animals were euthanized by exsanguination. Testes, epididymides, penile tissue and adrenal glands were carefully excised (Figure 3). Testes and adrenal glands were cleared of surrounding tissue, weighed and recorded. Fresh tissue samples were either processed immediately or stored under appropriate conditions for subsequent analyses.

**FIGURE 3:**
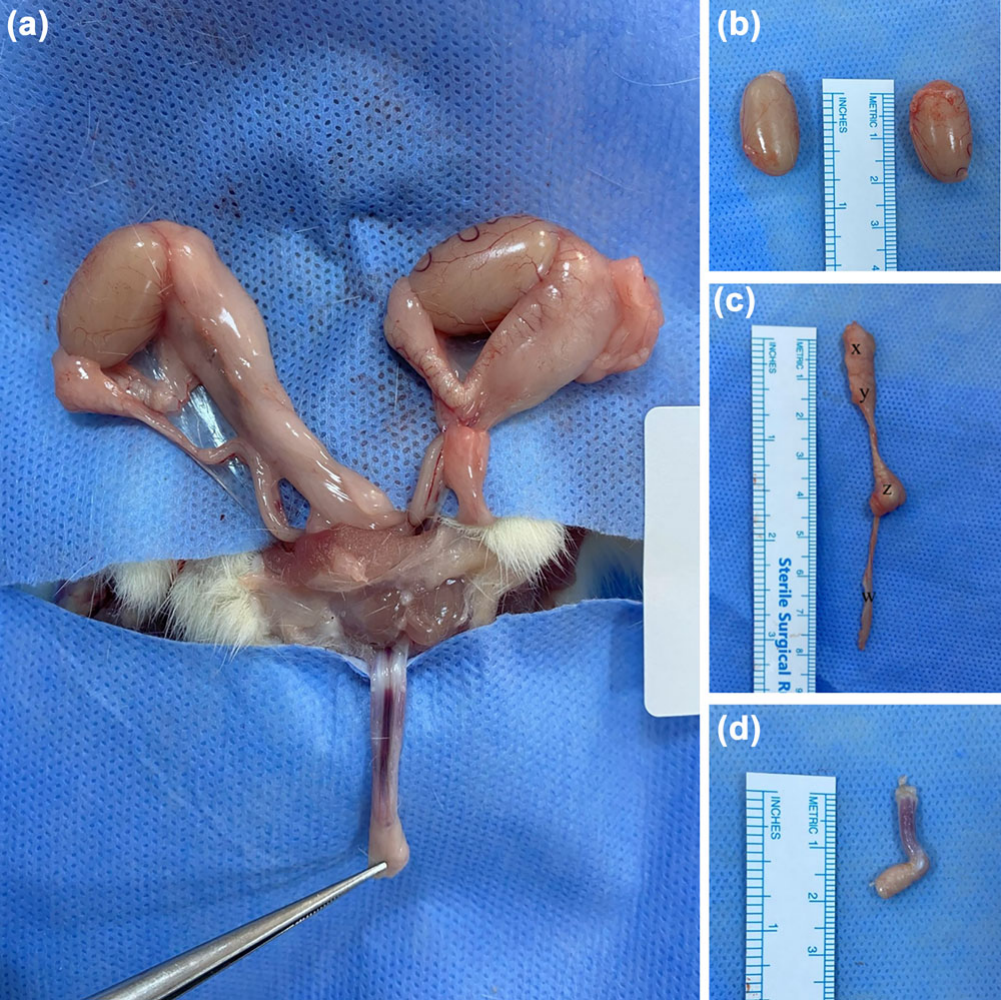
Isolated male reproductive tissues. (a) Tissue collection procedure. (b) Isolated rat testis prepared for assessment of spermatogenic activity and histological analysis. (c) Dissected rat epididymis for evaluation of sperm concentration, motility and morphology. Anatomical regions are indicated as follows: x, caput epididymis; y, corpus epididymis; z, cauda epididymis; w, vas deferens. (d) Isolated rat penis prepared for histomorphological examination of erectile tissue architecture.

### 2.6 Determination of serum testosterone levels

Serum total testosterone concentrations were measured using a commercial ELISA kit (Elabscience, E‐OSEL‐R0003, TX, USA) following the manufacturer's instructions. Blood samples were allowed to clot at room temperature for 30 min and centrifuged at 1500 *g* for 15 min at 4°C to obtain serum, which was then stored at −80°C until analysis. The assay sensitivity was <0.07 ng/mL, with intra‐ and inter‐assay coefficients of variation <10%.

### 2.7 Histological analysis

Testicular and penile tissues were fixed in 10% neutral buffered formalin, dehydrated in graded ethanol, cleared in xylene and embedded in paraffin. Sections (5 µm) were cut using a rotary microtome and mounted on glass slides. Testicular sections were stained with haematoxylin and eosin (H&E), and penile sections with Masson's trichrome. Images were captured using a light microscope (Olympus BX53, Tokyo, Japan) with cellSens software (Evident Corporation, Tokyo, Japan).

Spermatogenesis was evaluated using the Johnsen scoring system (JSC), a semiquantitative method originally developed for human testicular biopsies (Johnsen, 1970) that has since been widely applied in experimental rat studies (Chang et al., 2023). The system assesses seminiferous tubules on a scale from 1 to 10, with 10 indicating complete spermatogenesis and 1 indicating the absence of germ cells.

To simplify the analysis and comparisons between groups and to focus on clear histopathological changes, the JSC scores were grouped into four simplified Johnsen score classification (SJSC) categories representing similar histological features: Category 1 (JSC 1–3), Category 2 (JSC 4–5), Category 3 (JSC 6–7) and Category 4 (JSC 8–10) (Ito et al., 2021) (Figure 4). This classification allowed the degree of spermatogenic impairment to be shown more clearly and the category distributions to be compared between the experimental groups. For each animal, a total of 50 seminiferous tubules were randomly selected from five different regions of the testicular section. Each tubule was assigned to one of the four SJSC categories according to its histological features. The number of tubules in each category was then determined for each animal, and the percentage distribution of the categories was calculated by dividing these values by the total number of tubules evaluated.

**FIGURE 4:**
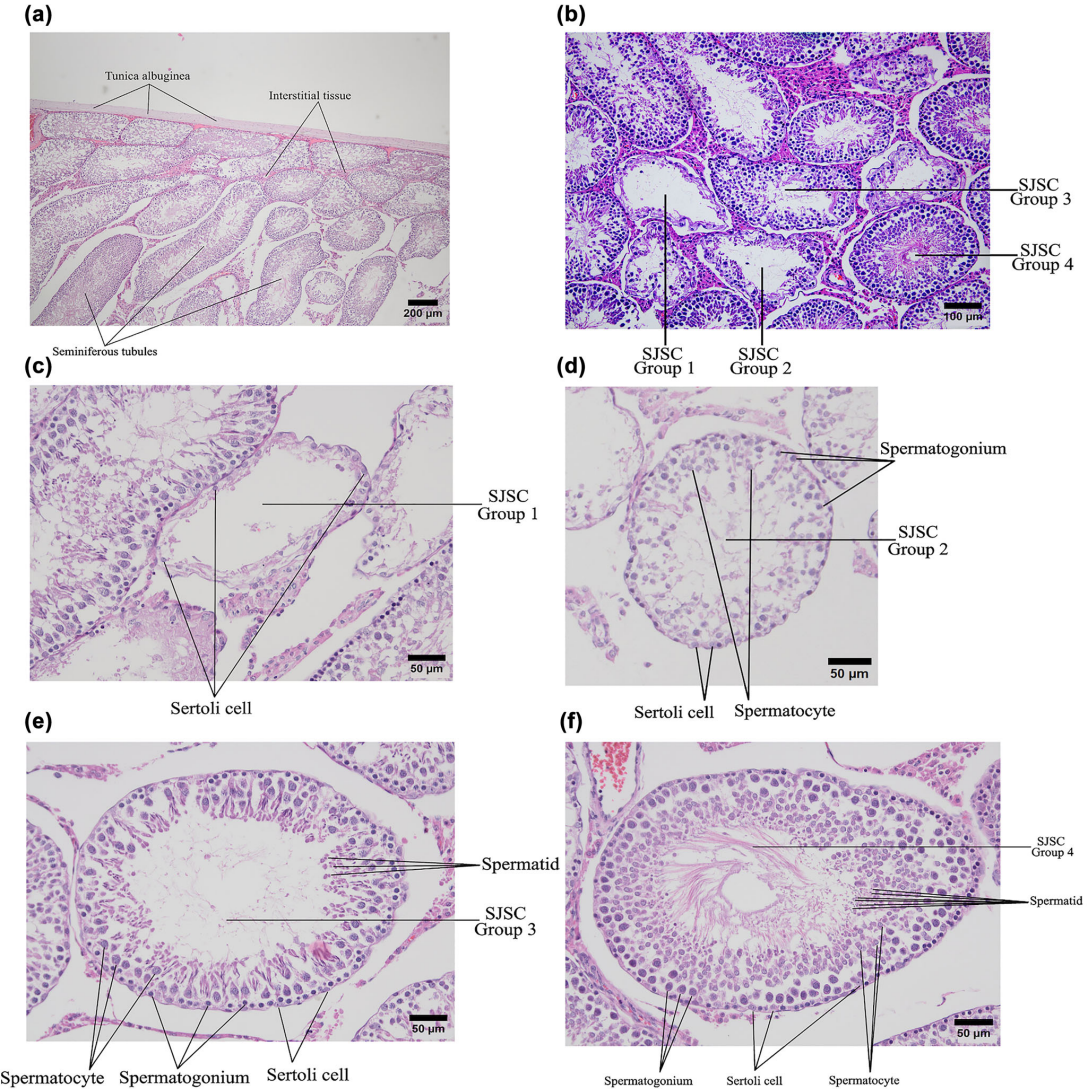
Histological evaluation of rat testis sections. (a) Cross‐sectional architecture of a control rat testis. (b) Representative section illustrating seminiferous tubules corresponding to all SJSC groups. (c) SJSC Group 1 (JSC 1–3): representative section from the S group, showing seminiferous tubules devoid of spermatocytes, indicative of severely impaired spermatogenesis. (d) SJSC Group 2 (JSC 4–5): representative section from the V group, showing seminiferous tubules containing spermatocytes but lacking spermatids. (e) SJSC Group 3 (JSC 6–7): representative section from the SV group, showing seminiferous tubules containing spermatids but lacking mature spermatozoa. (f) SJSC Group 4 (JSC 8–10): representative section from the C group, showing seminiferous tubules containing mature sperm cells, indicating preserved testicular architecture and active spermatogenesis. All images were captured at ×400 magnification. JSC, Johnsen Scoring System; SJSC, Simplified Four‐Group Johnsen Score Classification; C, control; S, sedentary; V, whole‐body vibration; SV, sedentary + whole‐body vibration.

A weighted mean SJSC score was also calculated for each animal. Higher mean SJSC scores indicated normal or near‐normal spermatogenesis, whereas lower scores indicated more severe spermatogenic impairment. Individual seminiferous tubules were treated as subsamples to characterize the spermatogenic status of each animal and were not considered independent statistical observations. All histological evaluations were performed by investigators who were blinded to the experimental group assignments.

### 2.8 Epididymal sperm collection and analysis

The caudal region of each epididymis was carefully excised, with the right epididymis used for sperm morphology and the left for concentration and motility assessments. Immediately after dissection, epididymal tissues were placed in Washing/Insemination medium (SIL‐Select Plus™, Lot No. FP15FL05, Beernem, Belgium), and sperm were released by small incisions and gentle pressure along the epididymal duct, followed by incubation for 30 min at 37°C.

Sperm concentration was measured using a Makler counting chamber. Motility was classified as progressive, non‐progressive or immotile and counted in 10 squares. For sperm morphology analysis, samples were smeared onto glass slides, air‐dried at room temperature and stained using the Rapi‐Diff II staining kit (Atom Scientific, Hyde, UK). Stained slides were examined under ×100 oil immersion lens with a light microscope (DM4000B, Leica, Wetzlar, Germany) and digital camera (DFC280 Plus, Leica), evaluating 200 sperm per sample for abnormalities in head, neck and tail morphology. To eliminate observer bias, all epididymal sperm assessments, including sperm concentration, motility and morphology, were conducted by an investigator blinded to the group allocations.

### 2.9 Analysis of erectile tissue

Penile sections stained with Masson's trichrome were examined under a light microscope. Three regions of interest were defined: the right corpus cavernosum, left corpus cavernosum and total cavernosal area. Images were captured at high resolution using a digital camera integrated with the microscope and analysed using ImageJ software (NIH, Bethesda, MD, USA). Areas corresponding to smooth muscle and collagen fibres were identified, and the smooth muscle‐to‐collagen ratio was calculated. Analyses were performed by a blinded observer.

### 2.10 Statistical analysis

Statistical analyses were performed using GraphPad Prism 10 (GraphPad Software Inc., Boston, MA, USA). The Shapiro–Wilk test was used to assess normality. Normally distributed data were analysed using one‐way analysis of variance (ANOVA), followed by Tukey's multiple comparisons test. Non‐normally distributed data were analysed using the Kruskal–Wallis test, followed by Dunn's multiple comparisons test with the two‐stage step‐up false discovery rate correction of Benjamini, Krieger and Yekutieli. Differences were considered statistically significant at *P* < 0.05.

## 3 RESULTS

### 3.1 Body and tissue weight

No significant differences in initial or final body weight were observed among the groups. The increase in body weight observed across all groups during the 28‐day intervention period was consistent with normal growth and maturation of adult male Wistar rats and was not specific to any experimental condition. Testicular weight tended to be lower in the S and SV groups than in the control group; however, these differences did not reach statistical significance. Because the final body weight variability appeared greater in the control group, potential outliers were assessed using Grubbs' test; no significant outliers were identified, indicating that the observed dispersion reflected normal biological variability within the group. Relative adrenal weight also did not differ significantly among groups (Table 1).

**TABLE 1:** Body weight and relative testicular and adrenal weights in the study groups.

	C	S	SV	V	*P*
Body weights (g)					
Initial	312.8 ± 20.6	312.0 ± 17.5	316.0 ± 12.1	314.5 ± 12.7	0.961
Final	394.5 ± 55.5	372.5 ± 31.8	372.7 ± 33.2	411.7 ± 31.3	0.160
Testis weight (g)	1.68 ± 0.19	1.39 ± 0.30	1.61 ± 0.13	1.48 ± 0.22	0.130
Relative testis weight (% of final body weight)	0.43 ± 0.06	0.37 ± 0.08	0.43 ± 0.05	0.36 ± 0.07	0.115
Relative adrenal weight (% of final body weight)	0.015 ± 0.003	0.012 ± 0.002	0.012 ± 0.002	0.012 ± 0.002	0.179

*Note*: Values are expressed as means ± SD (*n* = 8). Relative organ weight was calculated as (organ weight/final body weight) × 100. No significant differences were observed between groups (*P* > 0.05). Abbreviations: C, control; S, sedentary; SV, sedentary + whole‐body vibration; V, whole‐body vibration.

### 3.2 Testosterone Levels

Serum total testosterone levels did not differ significantly among the experimental groups. Pairwise comparisons showed no statistically significant difference between the sedentary and control groups (*P* = 0.068) or between the sedentary + WBV and sedentary groups (*P* = 0.058) (Figure 5).

**FIGURE 5:**
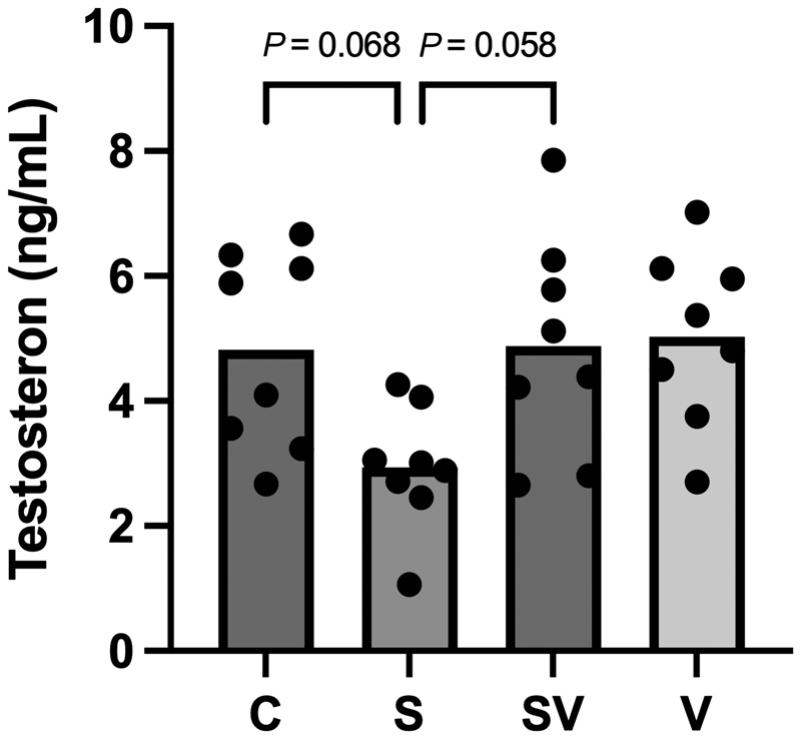
Serum testosterone levels in study groups. Serum testosterone concentrations in the control (C), sedentary (S), sedentary + whole‐body vibration (SV) and whole‐body vibration (V) groups after the 28‐day intervention period. *n* = 8 per group. Columns show mean values, and dots represent individual data points (*n* = 8 animals per group).

### 3.3 Testicular alterations associated with sedentary behaviour and WBV

The control group showed mostly normal spermatogenesis with preserved seminiferous tubule architecture, exhibiting the highest proportion of SJSC Group 4 and the highest overall SJSC score. In contrast, the sedentary group showed pronounced impairment of spermatogenesis, characterized by the highest proportion of SJSC Group 1, the lowest proportion of SJSC Group 4, and the lowest overall SJSC score among all groups. In the SV group, SJSC group distributions and SJSC scores did not significantly differ from controls. In the V group, both the percentage of SJSC Group 4 and the SJSC score were significantly lower than those of the control group (Figure 6).

**FIGURE 6:**
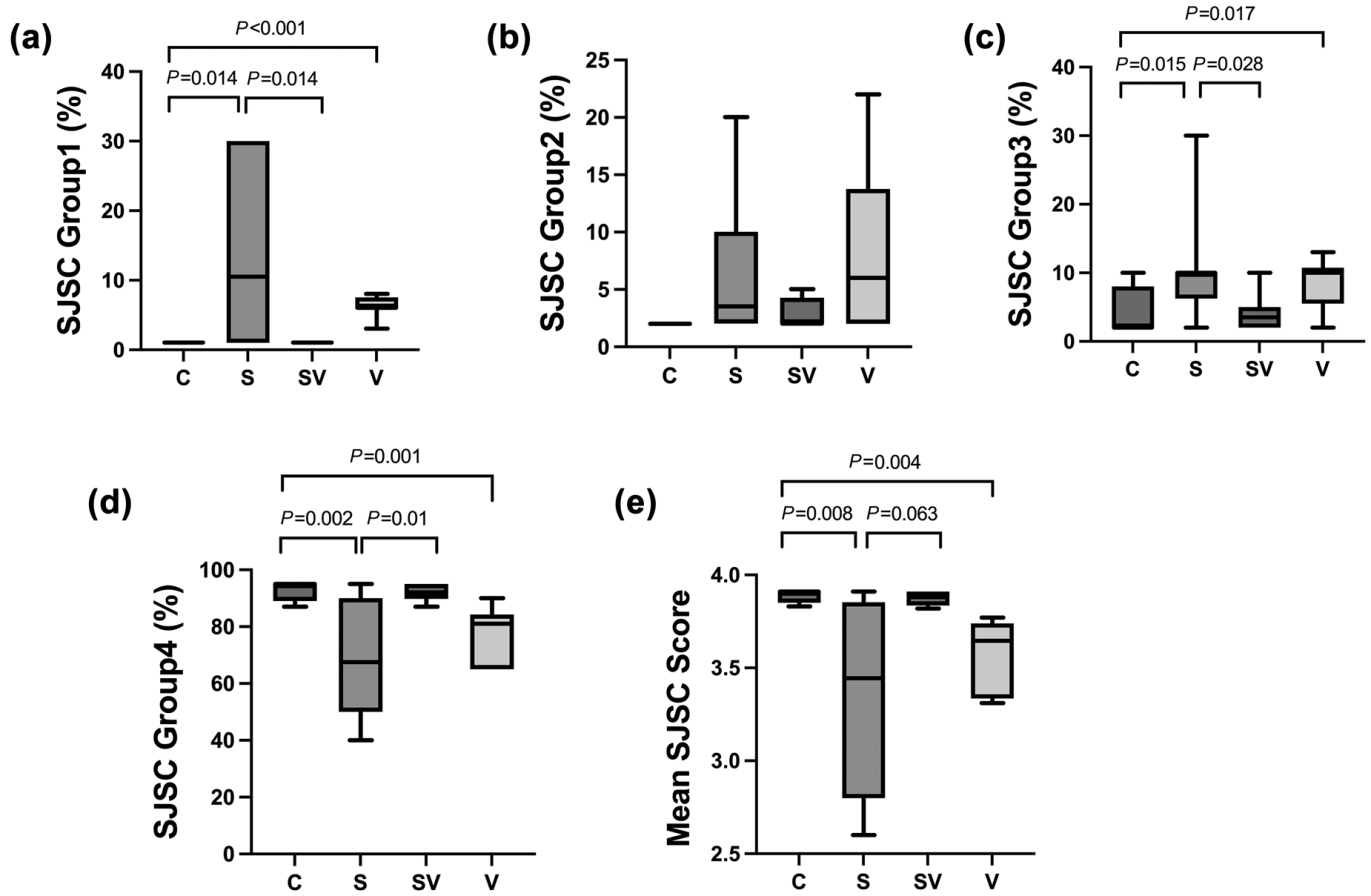
Distribution of simplified four‐group Johnsen score classification (SJSC) and overall SJSC scores among experimental groups. Relative distribution of SJSC Group 1 (a), SJSC Group 2 (b), SJSC Group 3 (c), SJSC Group 4 (d), and mean SJSC score (e) in the control (C), sedentary (S), sedentary + whole‐body vibration (SV) and whole‐body vibration (V) groups. SJSC Group 1 represents severely impaired spermatogenesis, whereas SJSC Group 4 is characterized by preserved spermatogenesis with intact seminiferous tubule architecture. Because individual seminiferous tubules were scored separately, tubules with different Johnsen score ranges could coexist within the same testis, reflecting heterogeneous spermatogenic damage.

### 3.4 Changes in sperm parameters associated with sedentary behaviour and WBV

Sedentary behaviour was associated with marked deterioration in sperm quality. Total sperm concentration was lowest in the S group and significantly lower than in the control group. In the SV group, sperm concentration was significantly higher than in the S group and did not significantly differ from controls. A similar pattern was observed for sperm motility parameters. Total and progressive motility were lowest in the sedentary group, whereas both measures were improved in the SV group compared with sedentary animals (Figure 7).

**FIGURE 7:**
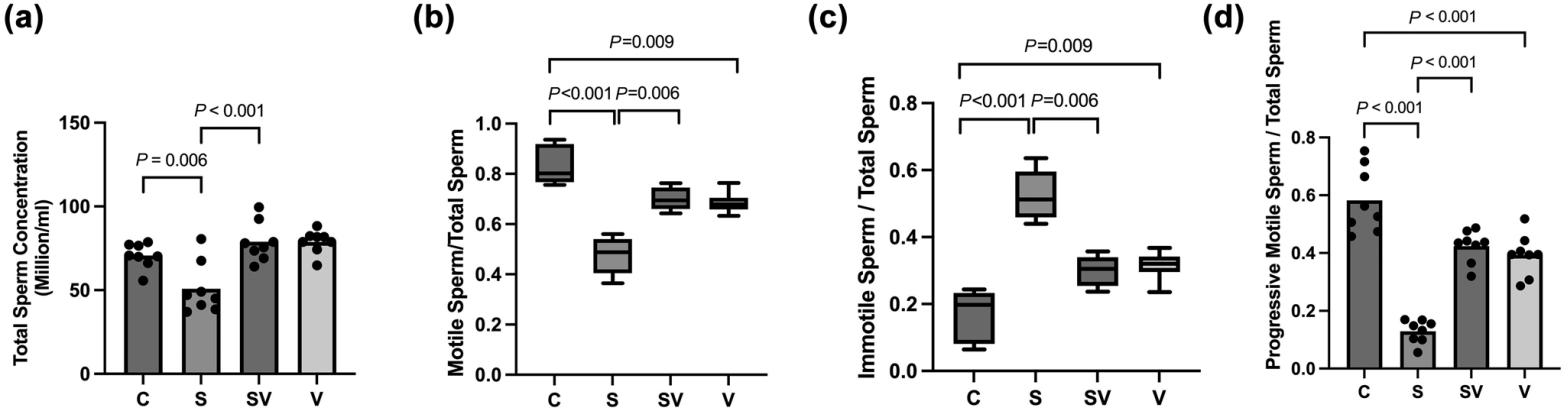
Sperm parameters across experimental groups. Total sperm concentration (a), total motile sperm ratio (b), immotile sperm ratio (c), and progressive motile sperm ratio (d) in the control (C), sedentary (S), sedentary + whole‐body vibration (SV) and whole‐body vibration (V) groups.

Head and neck abnormalities, as well as the overall proportion of abnormal sperm, were increased in the sedentary group relative to controls, whereas these abnormalities were reduced in the SV group. In contrast, the V group showed increased head and neck abnormalities and a higher proportion of total abnormal sperm compared with controls, suggesting that WBV alone may adversely affect sperm morphology in non‐sedentary animals (Figure 8).

**FIGURE 8:**
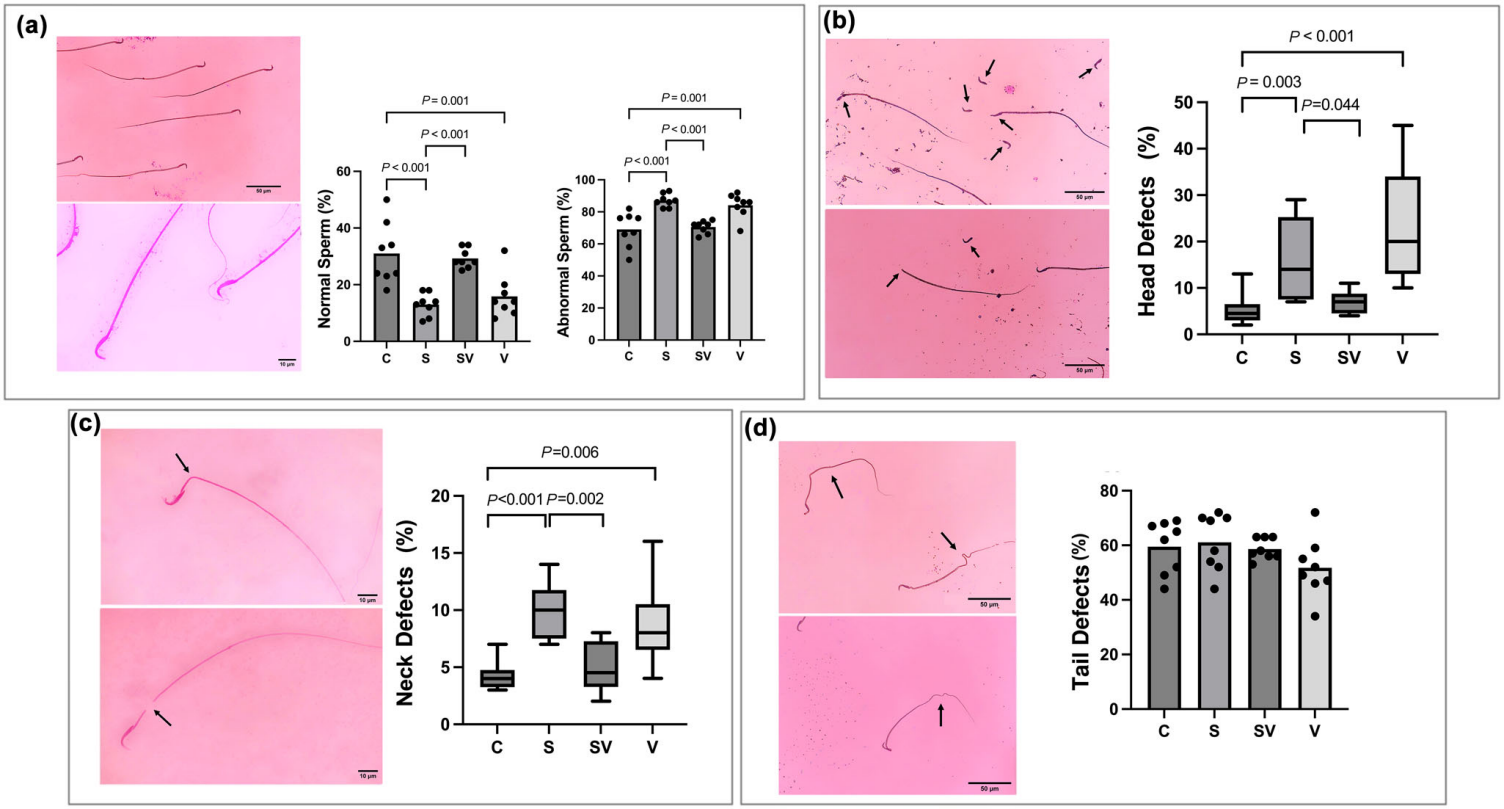
Sperm morphology parameters across experimental groups. (a) Representative micrographs and quantitative analysis of normal and abnormal sperm percentages. (b–d) Head defects (b), neck defects (c), and tail defects (d) in the control (C), sedentary (S), sedentary + whole‐body vibration (SV) and whole‐body vibration (V) groups.

### 3.5 Histological changes in penile tissue associated with sedentary behaviour and WBV

Sedentary behaviour significantly reduced the smooth muscle‐to‐collagen ratio compared with controls, indicating adverse cavernosal tissue remodelling. In the SV group, the ratio was significantly higher than in the sedentary group and approached control values, suggesting partial preservation of cavernosal tissue structure with WBV under sedentary conditions. The V group also showed a lower smooth muscle‐to‐collagen ratio than controls (Figure 9).

**FIGURE 9:**
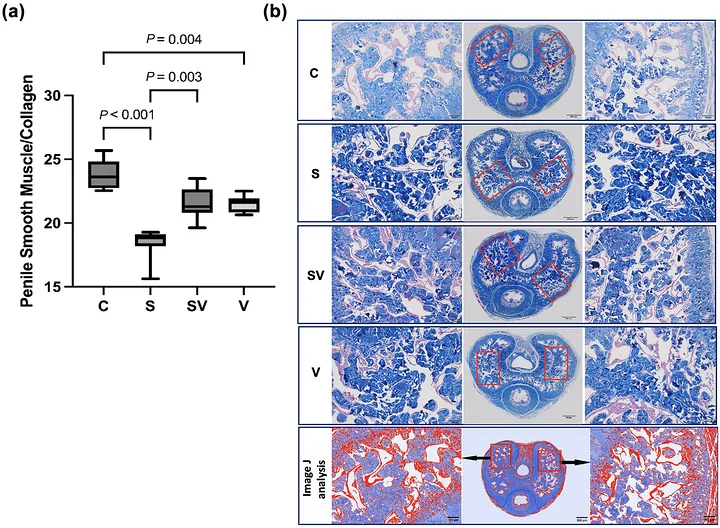
Smooth muscle‐to‐collagen ratio and histological features of penile tissue across experimental groups. (a) Quantitative analysis of the smooth muscle‐to‐collagen ratio in the corpus cavernosum of the control (C), sedentary (S), sedentary + whole‐body vibration (SV) and whole‐body vibration (V) groups. (b) Representative Masson's trichrome‐stained cross‐sections of penile tissue showing the right and left corpus cavernosum, as well as full transverse sections for each group. Smooth muscle fibres appear red and collagen fibres appear blue. An example of ImageJ‐based colour segmentation used for quantitative analysis is shown.

## 4 DISCUSSION

### 4.1 Overview of major findings

The present study investigated the effects of prolonged sedentary behaviour on male reproductive function and penile tissue structure in male rats and examined whether whole‐body vibration could attenuate these alterations. The findings indicate that experimentally induced sedentary behaviour was associated with impaired spermatogenesis, reduced sperm quality and adverse structural remodelling of penile tissue. Under sedentary conditions, WBV attenuated several of these alterations. However, in non‐sedentary animals, WBV alone was associated with unfavourable histological and sperm morphology findings, indicating that its biological effects may be strongly context‐dependent.

### 4.2 Validation of the sedentary behaviour model

Most previous animal studies involving immobilization or restraint were primarily designed to elicit physiological stress responses rather than to model sedentary behaviour. Consequently, these models are consistently associated with the activation of the hypothalamic–pituitary–adrenal axis, as evidenced by adrenal hypertrophy and elevated corticosterone levels (Arun et al., 2016).

In contrast, the reduced cage volume model employed in the present study was specifically selected to restrict spontaneous locomotor activity while minimizing stress exposure and has previously been shown not to increase corticosterone levels (Gungor‐Orhan et al., 2025; Marmonti et al., 2017). In the present study, circulating corticosterone concentrations were not directly measured. Although relative adrenal weight did not differ significantly among groups, this parameter should be interpreted only as an indirect indicator and does not exclude potential changes in hypothalamic–pituitary–adrenal axis activity (Table 1).

### 4.3 Sedentary effects on sperm and penile tissue

In our study, sedentary behaviour impaired spermatogenesis, as demonstrated histologically by lower Johnsen scores and a more unfavourable distribution of SJSC categories. This histological deterioration was accompanied by reduced sperm concentration, decreased total and progressive motility, and increased morphological abnormalities, particularly in the head and neck regions. Notably, this reproductive damage occurred in the absence of statistically significant changes in serum testosterone levels or testicular weight, suggesting that the pathological process may develop even without overt systemic endocrine suppression (Spaggiari et al., 2024). Although epidemiological evidence has linked sedentary lifestyle to lower testosterone levels and endocrine disturbances (Cai et al., 2025; Priskorn et al., 2016), our findings indicate that impaired spermatogenesis and declining sperm quality may constitute early markers of male reproductive dysfunction, even before systemic hormonal changes become evident.

Previous experimental studies using restraint‐based stress models have reported variable effects on spermatogenesis, ranging from minimal alterations to substantial impairment (Arun et al., 2016; Mehfooz et al., 2018). However, those studies involved direct stress induction. In contrast, our findings show that sustained reduction in spontaneous movement alone, without explicit stress, is sufficient to disrupt spermatogenic integrity and sperm quality.

It should be noted that the 28‐day intervention period did not encompass a complete spermatogenic cycle in rats. The seminiferous epithelial cycle lasts approximately 12.9 days, whereas complete spermatogenesis requires approximately 50–60 days (Clermont, 1972). Consequently, some sperm evaluated at sacrifice may have initiated differentiation before the intervention, whereas germ cells exposed during the early phase of the intervention may not yet have completed maturation and reached the epididymis. Nevertheless, significant alterations in spermatogenesis and sperm quality were already evident after this relatively short intervention period. Future studies employing longer intervention periods are warranted to determine whether more prolonged sedentary exposure results in a greater magnitude or a different pattern of reproductive alterations.

Sedentary conditions also affected penile erectile tissue. A reduction in the smooth muscle‐to‐collagen ratio suggested loss of smooth muscle within the corpus cavernosum and fibrosis‐compatible structural remodelling of penile tissue. Such changes may weaken the veno‐occlusive mechanism and may have adverse implications for erectile physiology (Nehra et al., 1998). Therefore, prolonged sedentary exposure appears capable of simultaneously impairing both sperm production and erectile tissue integrity.

Evidence examining the relationship between sedentary behaviour and male reproductive function in humans remains limited and methodologically heterogeneous. Foucaut et al. (2019) reported that sedentary behaviour was not an independent risk factor for male infertility and emphasized the difficulty of disentangling sedentary exposure from other lifestyle‐related variables. Most human studies have focused on semen parameters rather than fertility outcomes and have yielded inconsistent findings (Sterpi et al., 2025). Some investigations reported no association between sedentary behaviour and sperm concentration (Gaskins et al., 2014; Sun et al., 2019), whereas others observed reduced semen volume or sperm concentration with prolonged television viewing or extended periods of inactivity (Gaskins et al., 2015; Hjollund et al., 2002; Priskorn et al., 2016). In contrast, sperm motility has generally shown little or no association with sedentary behaviour across studies (Gaskins et al., 2015; Hjollund et al., 2002; Priskorn et al., 2016; Sterpi et al., 2025). Data regarding sperm morphology remain scarce; however, Al‐Bayyari (2017) reported that prolonged television viewing and extended wireless phone use were significantly associated with decreased percentages of normal sperm morphology.

These heterogeneous findings highlight key methodological limitations in human research, including reliance on self‐reported sedentary exposure, variability in definitions of sedentary behaviour, and insufficient control of confounding factors such as adiposity, metabolic status, occupational posture and environmental conditions. Furthermore, different sedentary domains, such as desk‐based work, prolonged driving or screen exposure, may differ in their behavioural and physiological characteristics, contributing to variability in biological outcomes across studies. Controlled experimental models help isolate the direct biological effects of sedentary behaviour while minimizing environmental confounding, thereby providing a clearer framework for understanding its impact on male reproductive function and erectile tissue.

An additional consideration is that sedentary behaviour in quadrupedal rodents is not entirely equivalent to sedentary behaviour in humans. In humans, sedentary behaviour is typically characterized by prolonged sitting or reclining (Tremblay et al., 2017), whereas rats remain in a quadrupedal posture during periods of inactivity. Consequently, the mechanical and haemodynamic consequences of reduced movement are unlikely to be identical between species. Although the reduced‐cage model effectively limits spontaneous locomotor activity and enables investigation of the biological consequences of sedentary behaviour under controlled conditions, these species‐specific differences should be considered when translating the present findings to humans. Furthermore, although the fundamental histological organization of the testis and penile erectile tissue is comparable between rats and humans, important species‐specific anatomical differences exist and should be considered when interpreting the translational relevance of the present findings (Hsu et al., 2005; Knoblaugh et al., 2018)

### 4.4 Role of whole‐body vibration under sedentary conditions

WBV applied during prolonged sedentary conditions may attenuate several adverse changes in male reproductive and penile tissues. In the Sedentary + WBV group, spermatogenesis was better preserved than in sedentary controls, with higher sperm concentration and motility and fewer morphological abnormalities. In parallel, the smooth muscle‐to‐collagen ratio within the corpus cavernosum was higher than that in the sedentary group and approached control values, suggesting attenuation of sedentary‐related structural deterioration.

Importantly, WBV did not exert uniformly beneficial effects under all conditions in the present study. Previous experimental evidence indicates that high‐dose or prolonged exposure to WBV may adversely affect male reproductive tissues (Krajnak et al., 2022; Zarei et al., 2022). In the current study, WBV applied to non‐sedentary animals was associated with impaired spermatogenesis, less favourable sperm motility and morphology, and a lower cavernosal smooth muscle‐to‐collagen ratio compared with controls. Collectively these results indicate that the biological effects of WBV are context‐dependent rather than universally protective.

Because oxidative stress, inflammation, tissue perfusion and circulating corticosterone were not directly assessed in the present study, the following discussion is intended to generate hypotheses rather than provide definitive mechanistic explanations. Under sedentary conditions, characterized by reduced basal circulation and diminished neuromuscular activation, WBV may act as a compensatory mechanical stimulus that partially offsets the biological consequences of reduced movement. In this setting, WBV may improve local blood flow (Betik et al., 2021; Johnson et al., 2014), enhance tissue oxygenation (Robbins et al., 2012) and reduce oxidative stress (Liu et al., 2016) in tissues. By contrast, when applied to animals without movement restriction, WBV may exceed adaptive capacity and function as a mechanical stressor, potentially activating inflammatory and oxidative pathways that impair tissue structure and spermatogenesis.

Although serum testosterone levels were not significantly different among groups, the observed between‐group differences approached statistical significance. Given the inherent biological variability of circulating testosterone in rats and the statistical power available for this endpoint, the present findings should not be interpreted as definitive evidence of the absence of endocrine effects.

Another important finding was the preservation of the smooth muscle‐to‐collagen ratio in the corpus cavernosum in sedentary animals exposed to WBV, indicating attenuation of fibrosis‐related remodelling associated with sedentary behaviour. Because cavernosal smooth muscle integrity is central to the veno‐occlusive mechanism required for penile erection, this structural preservation provides a plausible explanation for the observed tissue‐level effects of WBV. Previous studies have shown that WBV can enhance skeletal muscle perfusion, stimulate angiogenesis and improve extracellular matrix remodelling (Betik et al., 2021; Roberts et al., 2021; Weinheimer‐Haus et al., 2014). Experimental evidence also suggests that low‐intensity vibration may reduce collagen accumulation and limit fibrotic changes (Corbiere et al., 2018). Together, these observations provide a plausible hypothesis that WBV may help preserve erectile tissue architecture under sedentary conditions. However, the biological mechanisms underlying these findings were not directly investigated in the present study.

Several limitations should be considered when interpreting these findings. First, the reduced‐cage model may not fully replicate the complexity of sedentary behaviour in humans. Second, erectile function was evaluated histologically rather than through direct physiological functional testing, and fertility outcomes were not assessed. Third, blood and sperm parameters are subject to considerable biological variability, including diurnal and individual differences. Without baseline measurements, and given the cross‐sectional nature of the group comparisons, an uneven distribution of this variability across groups cannot be entirely ruled out. Fourth, potential mechanistic pathways, including circulating corticosterone, oxidative stress, inflammation, endothelial signaling and tissue perfusion, were not directly examined. Consequently, the mechanistic interpretations presented in this study should be considered hypothesis‐generating. In addition, indirect calorimetry was not performed to characterize the metabolic profile associated with the sedentary model. Future studies should clarify these biological mechanisms, incorporate metabolic assessments to further characterize the sedentary model, and determine the optimal WBV parameters required to achieve beneficial effects while avoiding potential adverse outcomes.

### 4.5 Conclusion

This study contributes to the limited literature on sedentary behaviour and male reproductive as well as penile tissue health. In this rat model, prolonged sedentary behaviour impaired spermatogenesis, reduced sperm quality and induced adverse structural changes in penile tissue. WBV applied under sedentary conditions attenuated several of these alterations, suggesting a context‐dependent beneficial role in this specific setting. However, WBV alone was associated with unfavourable reproductive and penile tissue findings, indicating that its effects are context‐dependent. Further mechanistic and clinical studies are needed to clarify these findings and determine whether WBV may have a role in sedentary behaviour‐related male reproductive impairment and penile tissue remodelling.

## AUTHOR CONTRIBUTIONS

Mujdat Ayva: Conceptualization, methodology, investigation, writing—original draft. Senay Akin: Methodology, investigation, project administration, writing—original draft. Irem Gungor Orhan: Methodology, formal analysis, investigation. Neslihan Coskun Akcay: Methodology, investigation, writing—review and editing. Berkay Ozerklig: Methodology, formal analysis, investigation. Kemal Kosemehmetoglu: Methodology, investigation, visualization, writing—review and editing. Mesut Altan: Conceptualization, methodology, supervision. Haydar A. Demirel: Conceptualization, methodology, supervision, project administration, writing—review and editing. Fazil Tuncay Aki: Conceptualization, supervision, project administration. All authors critically revised the manuscript. All authors have read and approved the final version of this manuscript and agree to be accountable for all aspects of the work in ensuring that questions related to the accuracy or integrity of any part of the work are appropriately investigated and resolved. All persons designated as authors qualify for authorship, and all those who qualify for authorship are listed.

## CONFLICT OF INTEREST

None declared.

## GENERATIVE AI STATEMENT

Microsoft Copilot (GPT‐5) was used solely for language editing and improvement of manuscript readability. All scientific content, interpretations and conclusions were generated and verified by the authors.

## Supporting information



Supporting information
Table S1. Complete dataset of individual rat measurements from all experimental groups.

## Data Availability

All data generated and analysed during this study are available in the Supporting Information files.
